# The Diagnosis and Separation of Peritoneal Adhesions around the Displaced Uterus and Ovaries

**Published:** 1888-02

**Authors:** 


### *THE DIAGNOSIS AND SEPARATION OF PERITONEAL ADHESIONS AROUND THE DISPLACED UTERUS AND OVARIES.*

Schultz (*Zeitschrift f. Geburtsh. u. Gynäkol.*, Bd. xiv., Heft 1,) introduces a paper on this subject, with the statement that in most cases of fixation of the retroflexed uterus, the displacement occurs first, the organ being imprisoned in its abnormal position by a subsequent attack of peritonitis. The adhesions can usually be felt by the finger, introduced into the rectum or vagina. In obscure cases, the uterine canal may be dilated, and the finger may be introduced to the fundus, so as to steady the organ, while the hand over the abdomen is enabled to draw the fundus forward, and thus to estimate the strength of the cicatricial bands. A sound may be substituted for the finger within the rectum, rectal palpation being practiced simultaneously. Anesthesia should be employed if the exact amount of mobility cannot be ascertained without it. The bladder and rectum having been emptied, the patient is placed in the lithotomy position, the examiner introduces his fore and middle fingers into the rectum, and presses them against the retroflexed fundus, at the same time resting the elbow of the corresponding arm upon his knee. If the fundus can be elevated as high as the promontory, it can then be grasped by the fingers of the other hand placed upon the abdomen. The site and extensibility of the adhesions can now be discovered; if these are slight, they will give way under the combined force of the two hands, while, if they are broader, they can be separated from the uterus by pressure of the external finger-tips.

Imprisoned ovaries may be freed by pressure made through the rectum; but when they are adherent to the posterior surface of the broad ligament, reposition is impossible. The finger within the rectum explores the surface of the prolapsed organ for a free edge, or an interspace between the ovary and its adhesions. If the latter can be found, the finger-tip is gently, but firmly, bored into it, when the adhesions will frequently give way. It may be necessary to repeat the operation before the ovary is completely freed. It must be effected very slowly and cautiously, the operator being especially careful not to make traction or pressure entirely upon the organ itself. The author adds that in several cases in which he practised this method ot reposition, he never observed any unfavorable symptoms following the operation. Absolute rest and ice applications are recommended immediately afterward. The uterus and appendages are kept in their normal position by means of a suitable pessary. The dangers of the operation are not great, provided that a careful diagnosis has been made previously in the manner indicated.

